# Superhydrophobic Ceramic Coatings by Solution Precursor Plasma Spray

**DOI:** 10.1038/srep24670

**Published:** 2016-04-19

**Authors:** Yuxuan Cai, Thomas W. Coyle, Gisele Azimi, Javad Mostaghimi

**Affiliations:** 1 Department of Mechanical and Industrial Engineering, University of Toronto, 5 King’s College Road, Toronto, ON, M5S 3G8, Canada; 2 Department of Materials Science and Engineering, University of Toronto, 184 College Road, Toronto, ON, M5S 3E4, Canada; 3 Department of Chemical Engineering and Applied Chemistry, 200 College Road, Toronto, ON, M5S 3E5, Canada

## Abstract

This work presents a novel coating technique to manufacture ceramic superhydrophobic coatings rapidly and economically. A rare earth oxide (REO) was selected as the coating material due to its hydrophobic nature, chemical inertness, high temperature stability, and good mechanical properties, and deposited on stainless steel substrates by solution precursor plasma spray (SPPS). The effects of various spraying conditions including standoff distance, torch power, number of torch passes, types of solvent and plasma velocity were investigated. The as-sprayed coating demonstrated a hierarchically structured surface topography, which closely resembles superhydrophobic surfaces found in nature. The water contact angle on the SPPS superhydrophobic coating was up to 65% higher than on smooth REO surfaces.

Superhydrophobic surfaces exhibit superior water repellent properties, and they have remarkable potential to improve current energy infrastructure^1^,^2^,^3^. Substantial research has been performed on the production of superhydrophobic coatings^4^,^5^. Recently, rare earth oxides were used as a means of creating hydrophobic surfaces^1^. However, these ceramic REO superhydrophobic coatings have not yet been adopted in many industries where potential applications exist due to the limited durability of the coating materials and the complex and costly fabrication processes.

Plasma spraying has been used extensively in industry because of its high deposition rate, capability of coating complex shapes, and the ability to process high melting temperature materials^6^. Because of the high melting temperature of rare earth oxides (over 2000 °C), plasma spray is an ideal candidate to fabricate REO superhydrophobic coatings. Conventional plasma spray uses powder with particle sizes ranging from 10 micron to 100 micron as the feedstock. The powder is entrained in a gas stream and injected into the plasma plume. Decreasing the particle size could potentially lead to nano-scale microstructures in the coating, which enhances coating properties such as strength, toughness and wetting behaviors^7^. However, in order for powder particles to have enough momentum to penetrate into the plasma plume, the carrier gas velocity needs to be increased as the average particle size is decreased. This reduces the plasma temperature and interferes with heat transfer from the plasma to the particles^8^. SPPS is a relatively new technique which circumvents this problem^9^. In SPPS a precursor salt of the coating material is dissolved in a solvent, and the solution is injected into the plasma instead of powder. During injection into the high velocity plasma, the solution breaks up into micron sized droplets. At a reasonable flow rate the solution droplets will have enough momentum to penetrate into the plasma without excessively cooling the plasma. Nano-particle precipitates form in the droplets as the solvent is vaporised, and may melt before reaching the substrate. Thus, it is possible to generate nano-featured coatings using SPPS.

In SPPS spraying conditions can be controlled in order to achieve the desired coating microstructure and functionality. The effect of each spraying parameter on the coating microstructure is often nonlinear. Here, we presented an extensive examination of the effects of different plasma spraying conditions on the coating microstructure and hydrophobicity. The solution used in the experiment was prepared by dissolving 99.999% ytterbium nitrate pentahydrate (Pangea International, Shanghai, China) in distilled water or a distilled water and ethanol mixture. The solution was deposited using an Axial III Series 600 plasma torch (Northwest Mettech Corp., BC, Canada) on stainless steel substrates (see Methods). Figure 1a is a schematic diagram of the spraying system, and Fig. 1b shows non-wetting water droplets on the coated surface. Twelve different spraying conditions were examined (Table 1). Scanning electron microscopy (SEM, Hitachi SU 3500, see Methods) was used to characterize the cross-sectional microstructures of the coatings. X-ray diffraction (XRD) was performed to determine the phase composition of the coating material (see Supplementary Fig. S1) using a Miniflex600 (Rigaku, MI, USA). The coating thickness, porosity, surface roughness, water contact angle and contact angle hysteresis for all spraying conditions were measured (see Supplementary Table S1).

## Results and Discussion

### Effect of standoff distance

**Figure 2:**
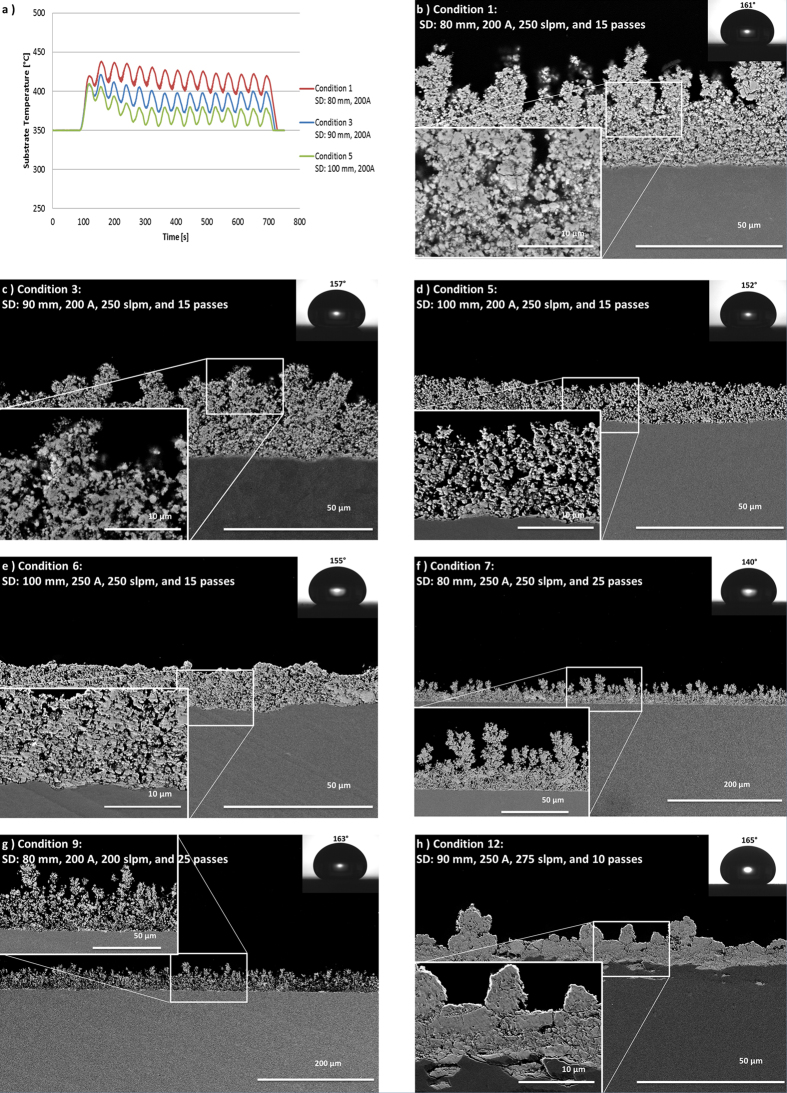
Temperature history and cross-sectional SEM images of selected deposition conditions. (**a**) Temperature history during depositions for conditions 1, 3 and 5. (**b**–**h**) SEM images of various spraying conditions.

From the results for conditions 1, 3, and 5 the effect of standoff distance (SD) can be investigated. Figure 2b–d show the cross sectional microstructures of coatings deposited with experimental conditions 1, 3, and 5 respectively and Fig. 2a shows the substrate temperature history for these three conditions. The coatings are porous and rough for all three conditions. Particles observed in the coatings have irregular shapes which is an indication of incomplete melting. This suggests that the coatings were formed mainly by the sintering of incompletely melted particles and aggregates of fully/partially decomposed precipitates from the droplets. Nano-particles were also observed in the coatings. Individual particles of this size would not have sufficient inertia to penetrate the gas boundary layer at the surface of the substrate. The thermophoresis force may have allowed these particles to pass through the boundary layer, or the nano-particles may have formed on the substrate from the condensation of vaporized material. When the standoff distance was increased while all the other parameters were held constant, an increase in the coating porosity, and decreases in the coating thickness and substrate temperature were observed. At long standoff distances, the plasma plume is cooled by the ambient air, resulting in cooler feedstock particles arriving at the substrate. The gas and particle velocities are also reduced at longer standoff distances. The combination of lower momentum and lower feedstock temperature at long standoff distances decreased the adhesion of the feedstock particles when they arrive at the substrate, which resulted in a reduction in the coating thickness and deposition efficiency. This agreed with the SEM images of deposits collected after a single torch pass, that show less material was deposited under condition 5 compared to conditions 1 and 3 (see Supplementary Fig. S2). The lower feedstock temperature at longer standoff distances also explains the increase in coating porosity, due to fewer molten droplets arriving at the substrate and less sintering of the fine particles after deposition. Water contact angles were higher on the coatings deposited at the short standoff distance, which correlates with a higher surface roughness (Supplementary Table S1 and Fig. S5).

### Effect of torch power

When the torch power was increased by increasing the arc current, a reduction in the coating porosity was observed (see Supplementary Table S1). Conditions 5 and 6 showed the most pronounced effect of torch power on coating porosity (Fig. 2d,e). The coating microstructure produced with a higher torch power (Fig. 2e) still contained many incompletely melted particles, but denser regions formed by molten splats were seen, resulting in a reduction in porosity of over 10% (Fig. 2d). In contrast with the reduction in coating porosity, the change in torch power has negligible effect on the coating thickness and water contact angle.

### Effect of number of torch passes

As the coating thickness increased during deposition, the roughness of the surface gradually increased, as shown in Fig. 2d. When the coating thickness exceeded approximately 20 microns, a feathery structure began to appear, as seen for both conditions 1 and 3 (Fig. 2b,c). This structure results from a shadowing effect, whereby small particles approaching the surface along a path which is not perpendicular to the surface deposit preferentially at high points on surface. Once the feathery structure begins to grow, the shadowing effect becomes more and more dominant. Therefore, a further increase in the number of torch passes during deposition leads to longer feathery structures, as shown in Fig. 2f,g. However, the exaggerated growth of the feathery structure was not beneficial to the hydrophobicity of the coating. The water contact angle was reduced to 140° for condition 7 (Fig. 2f). The water droplet penetrated into the spacing between the feathery structure columns, and the contact angle hysteresis increased to over 30°, an indication that the water droplets entered the Wenzel regime^10^.

### Effect of solvents

It has been reported that higher density coatings can be obtained by using a solvent consisting of a mixture of alcohol and water^8^. Adding ethanol to the solution can enhance heat transfer between the plasma and the feedstock solution^11^. Introducing alcohol into the solution also decreases the surface tension of the solution, improving the secondary breakup of the atomized solution in the plasma plume, resulting in smaller size droplets^8^. Smaller droplets require less total heat to evaporate the solvent and fully melt the solute, which should increase the density of the coating. This effect can be seen by comparing the porosity of the coatings deposited under conditions 2 and 7, and conditions 4 and 8. Thermogravimetric analysis and differential scanning calorimetry (TGA-DSC, NETZSCH STA F3, see Methods) were performed to examine the precipitate powders that resulted from drying both solutions (see Supplementary Fig. S4). From these results, the effect of ethanol on the nature of the initial precipitate is negligible; therefore the difference in coating structure must be attributed to the change in the break-up behaviour of the droplets and the difference in the enthalpy required vaporizing the two solvents.

### Effect of plasma velocity

The effect of plasma velocity was investigated in conditions 9 and 10. To decrease the plasma velocity compared to conditions 7 and 8, the total plasma gas flow rate was reduced from 250 slpm to 200 slpm and the nozzle size was increased from 10 mm to 13 mm. Figure 2g shows an SEM image of the coating deposited under condition 9. Among all the spraying conditions investigated, conditions 9 and 10 have the highest coating porosities (see Supplementary Table S1). Note that when the total plasma flow rate was reduced, the total torch power also decreased. However, conditions 9 and 10 have similar torch power, slightly higher enthalpy, and the same standoff distances as conditions 1 and 3 respectively, but higher porosity. The high coating porosity resulting from the lower gas flow rate agrees with previous findings of solution precursor flame-sprayed coatings^12^. When the plasma gas flow rate was decreased, the feedstock solution experienced less secondary breakup, resulting in larger droplets during the spraying process, which required more heat to melt. Also, the particles would have lower momentum before impacting the substrate. These factors together resulted in a lower packing density of the particles and higher porosity in the coating. The coating thickness for conditions 7 and 8 was similar to conditions 9 and 10. The water contact angle in conditions 9 and 10 is approximately 160°. Even though the coating was porous, the spacing between the feathery structures was reduced, and the water droplet remained in the Cassie regime during the contact angle measurement^13^ (see Supplementary Fig. S5).

### The optimized spraying condition

Based on the previous results, deposition of a dense superhydrophobic coating requires: a short standoff distance; a high arc current; a low number of torch passes; the addition of ethanol to the solvent; and a high plasma velocity. Conditions 11 and 12 include these requirements, and furthermore a lower feedstock flow rate was used to reduce the cooling of the plasma (Table 1). Much denser coatings were deposited under these conditions (Fig. 2h, and Supplementary Table S1). From the SEM images of the deposit formed from a single torch pass (see Supplementary Fig. S3), good coverage of the substrate was observed at the center of the plasma plume, and the deposit consisted mainly of pancake shaped splats, which indicate complete melting of the feedstock material. As Fig. 2h shows, the molten droplets can be seen to have flowed into a crater in the substrate surface formed by roughening of the substrate surface prior to deposition, which improves the mechanical interlocking between the coating and the substrate^14^. In between the dense regions, incompletely melted particles were observed. From the examination of the single torch pass, these particles resulted from feedstock that travelled at the perimeter of the plasma plume.

**Figure 3:**
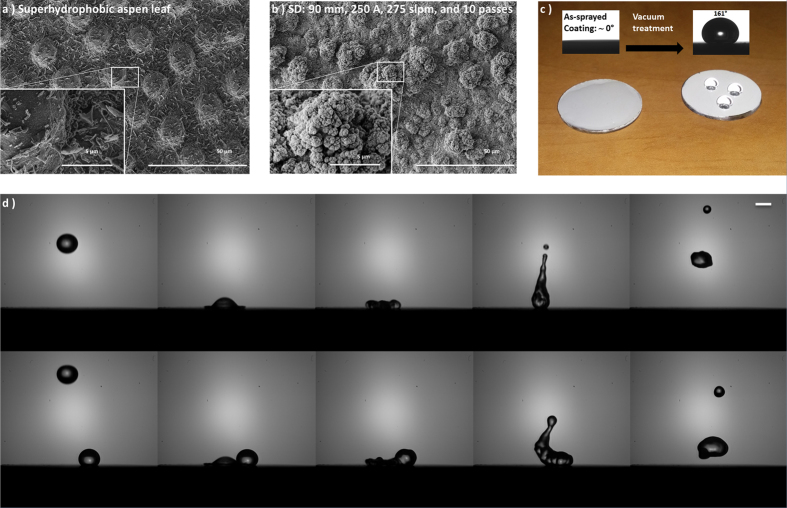
Topography and dynamic impacts of water droplets on the coated surface. (**a**) SEM image of the surface of a superhydrophobic quaking aspen leaf shows a hierarchically structured surface. (**b**) SEM image of the surface of a coating deposited under condition 12 exhibits a similar surface topography. (**c**) Change in wetting behaviours of the coating under various conditions. Sample size is 25.4 mm in diameter. (**d**) Dynamic impact of a single water droplet (top panel) and coalescence of 2 droplets (bottom panel) on the coated surface, scale bar 2 mm (see Supplementary Movies S1 and S2).

A uniform distribution of micro-scale irregular clusters ranging from 5 microns to 30 microns in size was observed on the surface of the coating (Fig. 3b and Supplementary Fig. S5). The clusters are agglomerates of individual particles less than 100 nm in diameter. This topography is consistent with the cross sectional images of the coating. The hierarchical structured top surface of the coating with a multi-scale roughness is very similar to the surface of superhydrophobic leaves in nature, such as the quaking aspen leaf (Fig. 3a). The as-sprayed coating surface was initially hydrophilic, but after vacuum treatment at 1 Pa for 48 hours the coating surface became superhydrophobic as shown in Fig. 3c. This transition is believed to be dependent on the surface oxygen-to-metal ratio^15^. The combination of the surface structure and the intrinsic hydrophobicity of the material gives the coating an excellent water repellent property (Fig. 3d).

A 0.1 m^2^ surface could be uniformly coated with an average coating thickness of 15 μm in less than 20 minutes in air using our laboratory set-up. This is over two orders of magnitude faster than other recently developed techniques for producing superhydrophobic surfaces such as laser ablation^16^,^17^ and physical vapor deposition^18^,^19^.

## Conclusions

In summary, we present a promising technique to fabricate superhydrophobic coatings using precursor solutions as feedstock in a plasma spray deposition process. It offers a fast, simple and low cost method to produce large area hydrophobic surfaces on a variety of substrates. The superhydrophobicity of the SPPS coatings results from the combination of the hydrophobic material and a hierarchically structured coating topography, which is similar to superhydrophobic surfaces found in nature.

## Methods

### Materials

99.999% Ytterbium nitrate pentahydrate (Pangea International, Shanghai, China) was dissolved in distilled water at 70% of the solubility limit of Yb(NO_3_)_3_ in water at 25 °C (167.3 g of ytterbium nitrate per 100 g of water) for conditions 1 to 6. For conditions 7 to 12, the solvent was replaced by a 50 wt% distilled water and 50 wt% ethanol mixture. The concentration of the ytterbium nitrate was reduced to 143.4 g of ytterbium nitrate per 100 g of solvent, because of the lower solubility of ytterbium nitrate in ethanol.

### Fabrication

For all experiments, a Mettech Axial III Series 600 plasma torch was used for coating deposition (Northwest Mettech Corp., North Vancouver, BC, Canada). The prepared solution was pumped through a 1.58 mm inner diameter polypropylene tubing by the liquid feedstock delivery system designed by Northwest Mettech Corp (North Vancouver, BC, Canada). The feedstock was injected into the plasma along the torch axis through a coaxial nozzle. The solution passed through the central 0.8 mm inner diameter capillary while Ar gas was fed through the annulus between the central capillary and the 3.25 mm ID outer tube at 10 liters per minute to atomize the liquid. The solution droplets were entrained in the plasma, where the solvent was evaporated and the precursor decomposed as the droplets were accelerated towards the substrate. The plasma was generated from a mixture of argon, nitrogen and hydrogen gas at various flow rates (Table 1). During deposition, the substrates were kept stationary, while the plasma torch was manipulated by a robot arm which moved in either a single linear pass to investigate the extent of melting of the deposition material or in a raster pattern in order to cover the whole surface of the substrates. The robot arm moved at a linear translation speed of 85 mm/s and the vertical step size in the raster pattern was 5 mm.

Type 304 stainless steel, 25.4 mm in diameter and 3.175 mm in thickness, was used as the substrate. The substrates were fabricated from cold-rolled steel by the supplier, and used with the surface finish as-received from the supplier. For conditions 11 and 12 the substrates were roughened by P120 silica grinding paper before deposition. All substrates were rinsed with tap water and dried in air, then placed in the substrate holder and pre-heated to 350 °C prior to the plasma spray deposition.

### Characterization

#### SEM

The as-sprayed coatings were sectioned by a precision diamond saw, IsoMet 5000 (Buehler, ON, Canada). The sectioned samples were then mounted in epoxy under a vacuum of 3000 Pa. A low viscosity epoxy (Jetset Epoxy, MetLab Corp, ON, Canada) was selected to allow the epoxy to penetrate into the pores of the cross section of the coating. The mounted coating’s cross section was subsequently polished using P320 silica grinding paper, followed in sequence by 45 μm, 15 μm, 6 μm and 3 μm diamond disks. Then 1 μm and 0.05 μm diamond suspensions were used, with ion milling (IM 4000, Hitachi, Japan) employed as the final stage of polishing. Between each polishing step, the surface was cleaned in an ultrasonic bath and dried by compressed air. An SEM (SU 3500, Hitachi, Japan) was used to characterize the surface and cross-sectional microstructures. To avoid charging effects in the SEM, specimens of the top surfaces of the coatings were sputter coated with gold and the polished cross sections were coated with carbon before observation. The images of the cross sections were used to quantify the coating thickness and the porosity of the coatings.

#### Coating thickness and porosity

For the coating thickness measurements, 8 different locations were randomly chosen from the SEM images at each condition, and the thickness at each location was measured. To calculate the coating porosity, SEM images were processed by ImageJ^20^. Due to the coating surface roughness, only the bottom half of the coating was examined. Each image was converted to 8 bits prior to calculation, the default threshold function Li was applied to convert the images into black and white, and subsequently the porosity values were calculated^21^ (see Supplementary Table S1).

#### XRD

X-ray diffraction was performed to determine the crystallographic phase of the coating material using Cu_Kα_ x-rays in a Miniflex600 (Rigaku, MI, USA). The coating was cut into 15 mm by 15 mm square specimens in order to fit the sample holder of the XRD machine. The measurements were performed over a range of 2θ angle from 15° to 105° with a scan speed 1°/min and a scan step of 0.02° (see Supplementary Fig. S1). The patterns obtained were compared with standard reference patterns (PDF card No.: 01-075-6635).

#### Wetting behavior measurements

We measured the water contact angle as previously described^17^. The water contact angle image was captured by a CCD camera (Sony XCD-SX900, Japan) with a horizontal microscope (Wild Heerbrugg 400076, Heerbrugg, Switzerland) at 5.8x magnification. Water droplets of 60 μL volume were used. For the CAH measurements, water is added or removed at a rate of 1.5 μL/min. The droplet was illuminated by a white-light projector from behind through a frosted glass. Image processing software, axisymmetric drop shape analysis (ADSA), was used to analyze the image of the water droplet in order to determine the water contact angle^22^. The dynamic impacts of water droplets on the coating were captured by a high speed camera FASTCAM SA5 (Photron, CA, USA) at 4000 frames per second.

#### TGA-DSC

Powders used in the TGA-DSC analysis were dried from the prepared solutions on a hot plate at 100 °C for 24 hours. Thermal analysis of the dried powders was analyzed by a NETZSCH STA 449 F3 (Netzsch, MA, USA). The test samples (~15 mg) were heated at a rate 10 °C/min up to 800 °C in flowing air at 35 kPa (see Supplementary Fig. S4).

#### Surface roughness

Surface images of the coatings were generated by the 3D-image capture function of the Hitachi TM 3000 SEM (Hitachi, Japan), and 3D surface profiles were generated by importing the SEM images into the 3D-Image Viewer software (Hitachi, Japan). From the 3D surface profile, surface roughness was analyzed by Fast Fourier Transform (FFT) from 5 Hz to 639 Hz at 20 locations per surface profile. Three surface profiles were examined for each sample, thus in total 60 locations were examined for each experimental condition (see Supplementary Table S1).

## Additional Information

**How to cite this article**: Cai, Y. *et al.* Superhydrophobic Ceramic Coatings by Solution Precursor Plasma Spray. *Sci. Rep.* **6**, 24670; doi: 10.1038/srep24670 (2016).

## Supplementary Material

Supplementary Information

Supplementary Movie S1

Supplementary Movie S2

## Figures and Tables

**Figure 1:**
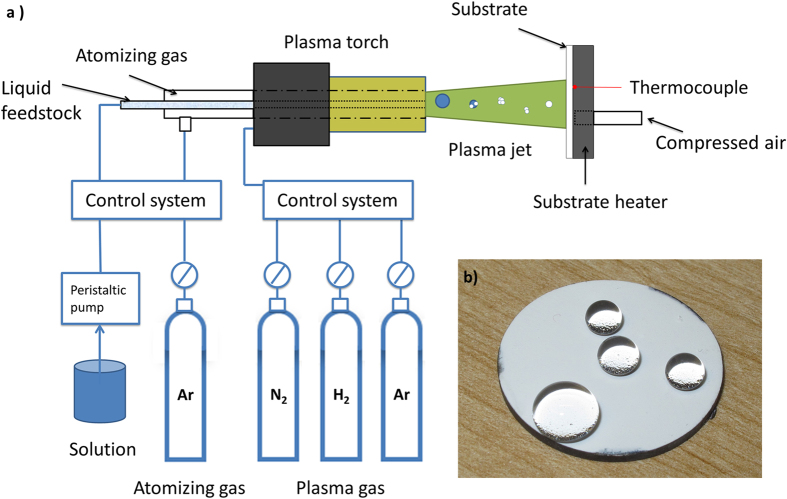
An overview of the SPPS process and the wetting behavior of the coated surface. (**a**) Schematic of the SPPS deposition system. (**b**) Water droplets of different sizes on the coated surface (Condition 1). The reflection at the bottom of the water droplets shows an air gap exists in between the droplet and the coating. Sample size is 25.4 mm in diameter.

**Table 1:** Summary of spraying conditions used in the depositions (mean ± SDV).

Condition	Standoff Distance [mm]	Ar [%]	N_2_ [%]	H_2_ [%]	Gas Flow Rate [slpm]	Arc Current Per Electrodes [A]	Power [kW]	Raster Passes	Feedstock Flow Rate [g/min]	Enthalpy [kJ/L]	Nozzle [mm]
1	80	15	80	5	250	200	125 ± 1	15	15	14.9 ± 0.1	10
2	80	15	80	5	250	250	141 ± 1	15	15	16.3 ± 0.6	10
3	90	15	80	5	250	200	122 ± 1	15	15	14.5 ± 0.5	10
4	90	15	80	5	250	250	143 ± 1	15	15	15.6 ± 0.5	10
5	100	15	80	5	250	200	129 ± 1	15	15	15.0 ± 0.1	10
6	100	15	80	5	250	250	143 ± 1	15	15	16.7 ± 0.2	10
7	80	10	80	10	250	250	157 ± 2	25	15	17.0 ± 0.3	10
8	90	10	80	10	250	250	159 ± 1	25	15	17.1 ± 0.5	10
9	80	10	80	10	200	250	124 ± 1	25	15	15.4 ± 0.2	13
10	90	10	80	10	200	250	124 ± 2	25	15	15.3 ± 0.3	13
11	80	14	72	14	275	250	170 ± 1	10	10	15.0 ± 0.5	8
12	90	14	72	14	275	250	171 ± 1	10	10	15.0 ± 0.1	8
