# Supplementary material for: Superhydrophobic Ceramic Coatings by Solution Precursor Plasma Spray
Source: Sci Rep. 2016 Apr 19;6:24670. doi: 10.1038/srep24670 (PMC4835754; doi:10.1038/srep24670)
Supplement: Supplementary Information [file srep24670-s1.doc]

**Supplementary Information**

**Superhydrophobic Ceramic Coatings by Solution Precursor Plasma Spray**

Y. Cai^*^, T.W. Coyle^†^, G. Azimi^#†^, and J. Mostaghimi^*^

Centre for Advanced Coating Technologies

^*^Dept. Mechanical and Industrial Engineering

^†^Dept. of Materials Science and Engineering

^#^Dept. of Chemical Engineering and Applied Chemistry

University of Toronto

**X-ray diffraction:**

The coating was cut into 15 mm by 15 mm square specimens in order to fit the sample holder of the XRD machine. The measurements were performed over a range of 2θ angle from 15° to 105°, and the patterns obtained were compared with standard reference patterns (PDF card No.: 01-075-6635). Figure S1 shows the selected XRD patterns of the coated surfaces. Peaks in the patterns match with the Yb_2_O_3_ reference at all peak locations. No additional peaks corresponding to the substrate were observed, which indicates that the coating material fully covered the substrate. The sharp peaks in the XRD patterns also indicate that the coating material is crystalline. The XRD patterns for all 12 spraying conditions are similar with only minor differences in some peak heights, indicating a random orientation of the crystallites in the coatings. Here only three conditions are shown.


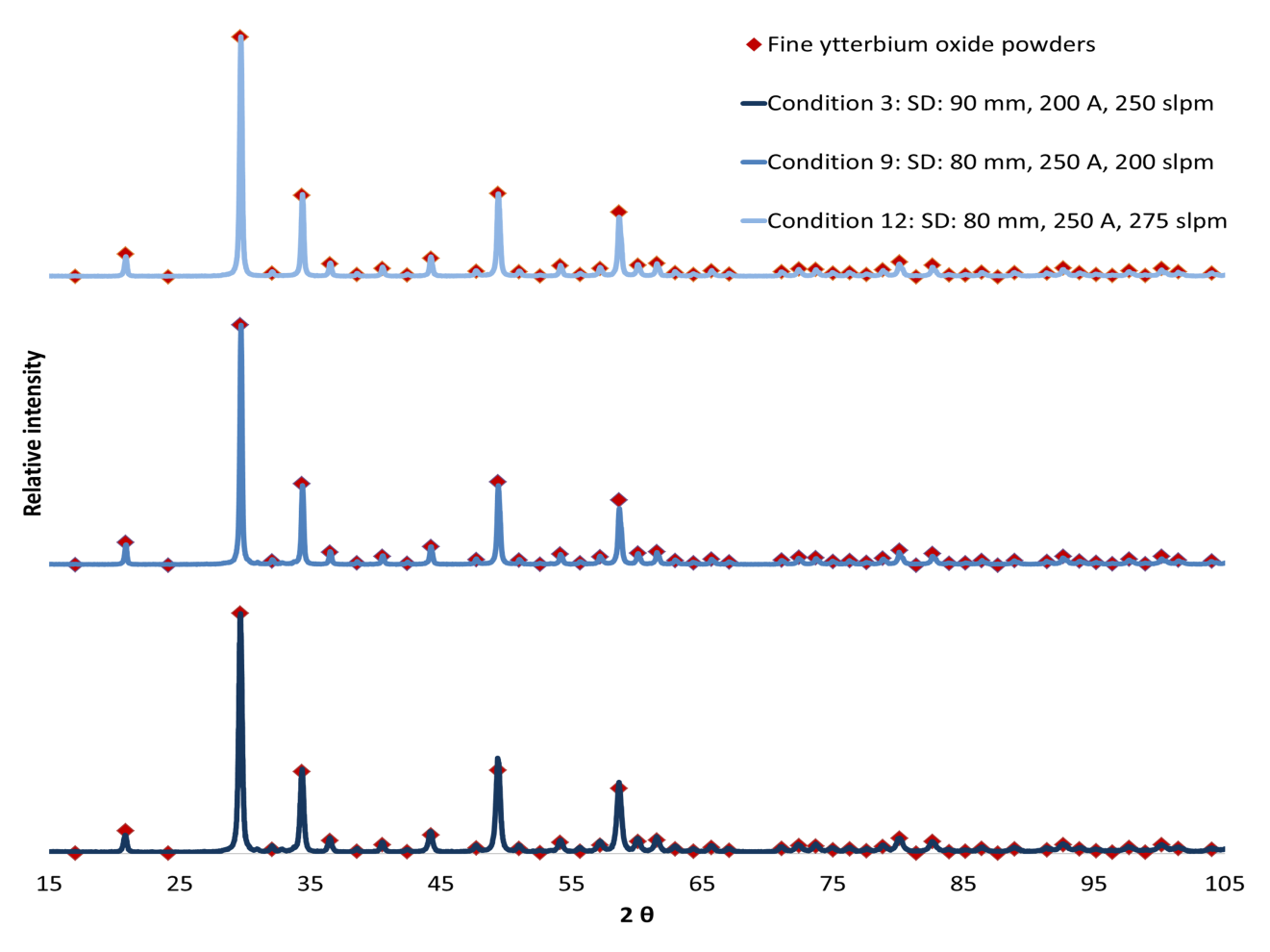


Figure S1: XRD patterns of selected spraying conditions.

**Summary of coating thickness, porosity, surface roughness, water contact angle, and CAH**

Table S1: Summary of coating thickness, porosity, surface roughness, water contact angle and CAH for each condition (mean ± SDV).

| **Condition** | **Thickness [μm]** | **Porosity [%]** | **Surface roughness Ra [μm]** | **Maximum Peak Height Rp [μm]** | **Water Contact Angle [°]** | **Contact Angle Hysteresis [°]** |
| --- | --- | --- | --- | --- | --- | --- |
| **1** | **34±7** | **35±2** | **1.7±0.1** | **8±2** | **161±1** | **4±1** |
| **2** | **31±10** | **31±3** | **1.8±0.1** | **8±2** | **162±2** | **4±2** |
| **3** | **26±6** | **36±1** | **1.3±0.5** | **7±3** | **157±1** | **3±2** |
| **4** | **24±5** | **32±2** | **1.2±0.1** | **6±1** | **157±1** | **2±1** |
| **5** | **13±2** | **44±4** | **0.9±0.1** | **4±1** | **152±1** | **6±2** |
| **6** | **13±3** | **32±4** | **0.8±0.2** | **4±1** | **155±2** | **4±1** |
| **7** | **34±15** | **27±2** | **2.6±0.7** | **16±9** | **140±1** | **32±3** |
| **8** | **33±16** | **28±3** | **2.2±0.1** | **9±2** | **140±1** | **30±2** |
| **9** | **37±13** | **59±4** | **2.6±1.0** | **11±6** | **163±2** | **5±2** |
| **10** | **31±12** | **61±4** | **1.6±0.5** | **9±4** | **160±2** | **4±1** |
| **11** | **11±6** | **12±3** | **1.1±0.3** | **5±2** | **163±2** | **4±1** |
| **12** | **12±11** | **17±4** | **0.9±0.3** | **4±2** | **165±2** | **2±1** |

**Single torch pass SEM images:**

Single torch pass depositions were performed to investigate the states of melting of the feedstock material. Figures S2 and S3 show some selected images of the single pass depositions. Since the axis of the torch passed across the middle of the circular substrate, more material was deposited close to the middle of the substrate than further away. Here images at both locations are presented.

In both Fig. S2 and Fig. S3, the deposits consist mainly of pancake shaped splats along the center of the torch pass, formed from fully melted particles upon impact. Nano-particles were also observed. A few incompletely melted particles and re-solidified particles were observed. All images in Fig. S2 are of deposits produced under the same arc current condition, but from different standoff distances. The substrate coverage decreased as the standoff distance increased from 80 mm to 100 mm. This indicates that at the 100 mm standoff distance a great portion of the feedstock material had cooled too much and did not stick on the substrate. The poor adhesion between the substrate and the coating material, and the lower coating thickness (lower deposition efficiency) observed for coatings deposited at the 100 mm standoff distance compared to the 80 mm and 90 mm distances are consistent with these single pass results.

Figure S3 shows the SEM images for conditions 6, 9 and 12. Comparing Figures S3a and S3b to Figures S2e and S2f, when the arc current was increased, the substrates were more completely covered by the deposit. The higher enthalpy of the plasma at higher current would be expected to increase the average particle temperature, and so improve the deposition efficiency. This is also consistent with the decrease in porosity observed in the coating cross-sections when the arc current was increased. Figures S3c and S3d show the effect of lower plasma flow rate. The least amount of feedstock material coverage was found in these two images, which is also in line with our observations in the cross-sectional SEM images. Finally, Figure S3e and S3f show the images of the deposit formed by single torch pass condition 12. Even though this condition has the lowest feedstock flow rate, the best coverage of the substrate was observed. Even at the periphery of the plasma plume, many pancake shaped splats were observed.

Recall that the cross sectional SEM images for coatings deposited under conditions 1, 3 and 5 showed many particles having irregular shapes (Figs. 1b, 1c, and 1d). This indicated that much of the feedstock material was not fully melted when it arrived at the substrate. The coating was formed in large part by the sintering of incompletely melted particles and aggregates of fully and/or partially decomposed precipitates from the droplets. Very fine nano-scale particles were also found in the cross sectional microstructures, which is consistent with the single torch pass observations. However, the deposits formed by a single torch pass consist predominantly of pancake shaped splats both at the center of the deposit and at the periphery of the deposit. Careful examination of the cross section images reveals that at the substrate-coating interface, evidence of pancake shaped splats can be seen, but only for the first few layers adjacent to the substrate. Apparently the incompletely melted particles do not stick well to the bare substrate on the first torch pass, so that pancake shaped splats formed from the fully melted droplets are primarily what is observed. For the subsequent torch passes, incompletely melted particles were able to adhere to the previously deposited splats and sintered during the subsequent torch passes.


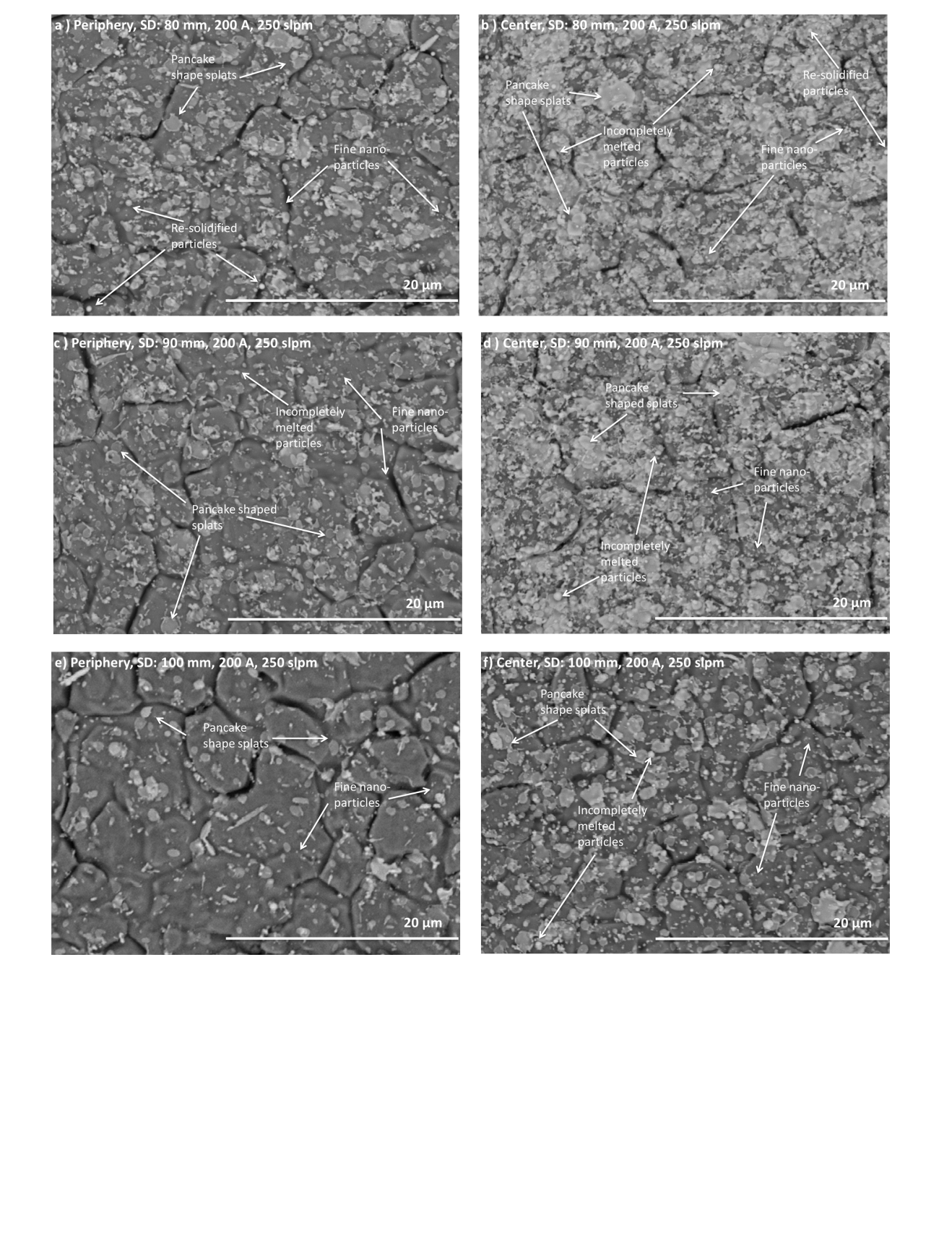


Figure S2: Single torch pass SEM images of top surfaces. a, c, and e show the SEM images at the periphery of the torch pass for conditions 1, 3 and 5 respectively. b, d, and f show the SEM images at the center of the torch pass for conditions 1, 3 and 5 respectively. The dark lines on the SEM images are due to the roughness of the cold rolled stainless steel.


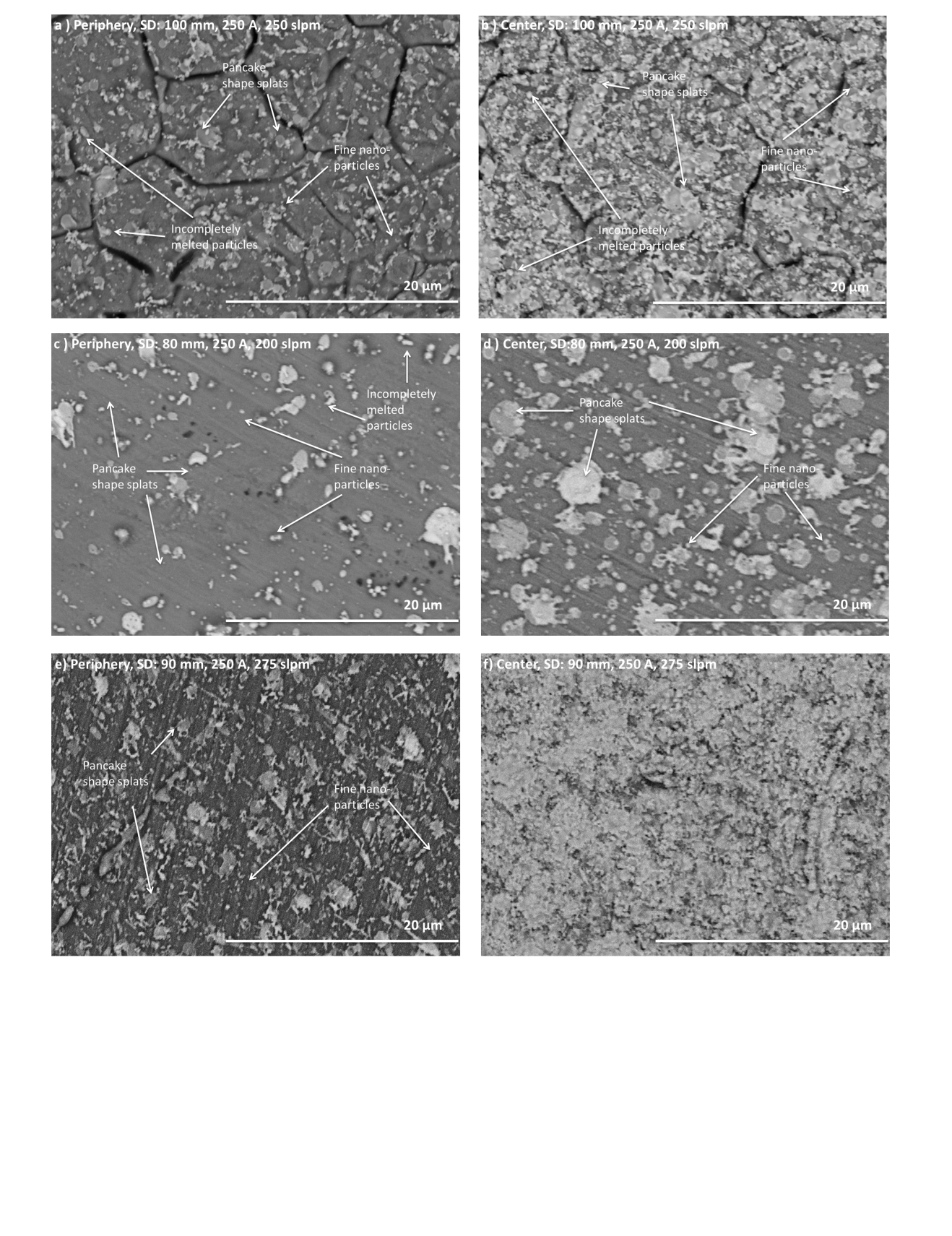


Figure S3: Single torch pass SEM images of top surfaces. a, c, and e show the SEM images at the periphery of the torch pass for conditions 6, 9 and 12 respectively. b, d, and f show the SEM images at the center of the torch pass for conditions 6, 9 and 12 respectively.

**Effect of solvents on the dried precipitate powder:**

After the atomized feedstock is introduced into the plasma, the solvent evaporates, and the solute will precipitate. Here we examine if the addition of alcohol to the solvent affects the subsequent decomposition behavior of the precipitates. Solutions formed using pure water as solvent and 50% water / 50% ethanol as solvent were dried on a hot plate at 100°C, and the dried precipitate powders were investigated by TGA-DSC

The DSC curves (Fig. S4a) reveal the thermal events of the dried powders during heating at 10°C/min. Between 200 °C to 285 °C, the powders dried from the water/ethanol mixture demonstrated a slightly more exothermic reaction than the powders obtained from the pure water solution, and the opposite happened from 285°C to 310°C. The net heats released by these two powders are nearly the same. The weight loss curves of these two powders during heating are also very close (Fig. S4b). From these results, we can conclude that adding ethanol to the solution has negligible effect on the decomposition behavior of the precipitates.


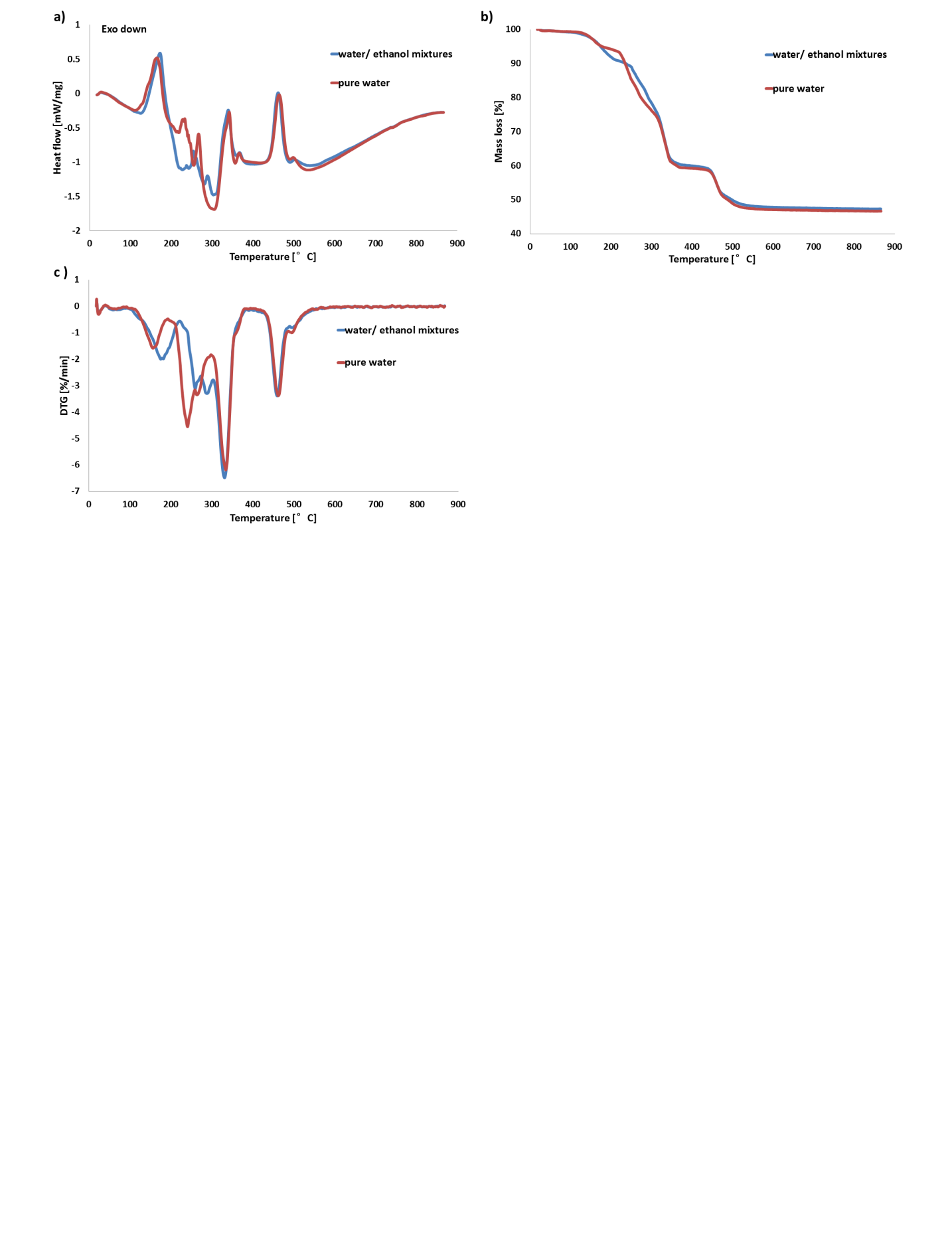


Figure S4: TGA-DSC analysis of the dried precipitate powders from the two solutions. a, b and c are DSC, TG and DTG curves respectively.

**Surface SEM images:**

Figure S5 summarized the topographies of the coatings generated from different spraying conditions. These surface SEM images are consistent with the cross-sectional images that were shown in Figure 2. Feathery structures can be observed for conditions 1 and 3 (Figs. S5a and S5b), and condition 5 showed a relatively smooth surface (Fig. S5c). Larger feathery structures can be observed for conditions 7 and 9, resulting in higher roughness surface profiles. Condition 11 resulted in a cauliflower-like surface structure (Fig. S5f) in which irregular clusters can be observed, similar to the surface of the aspen leaf.


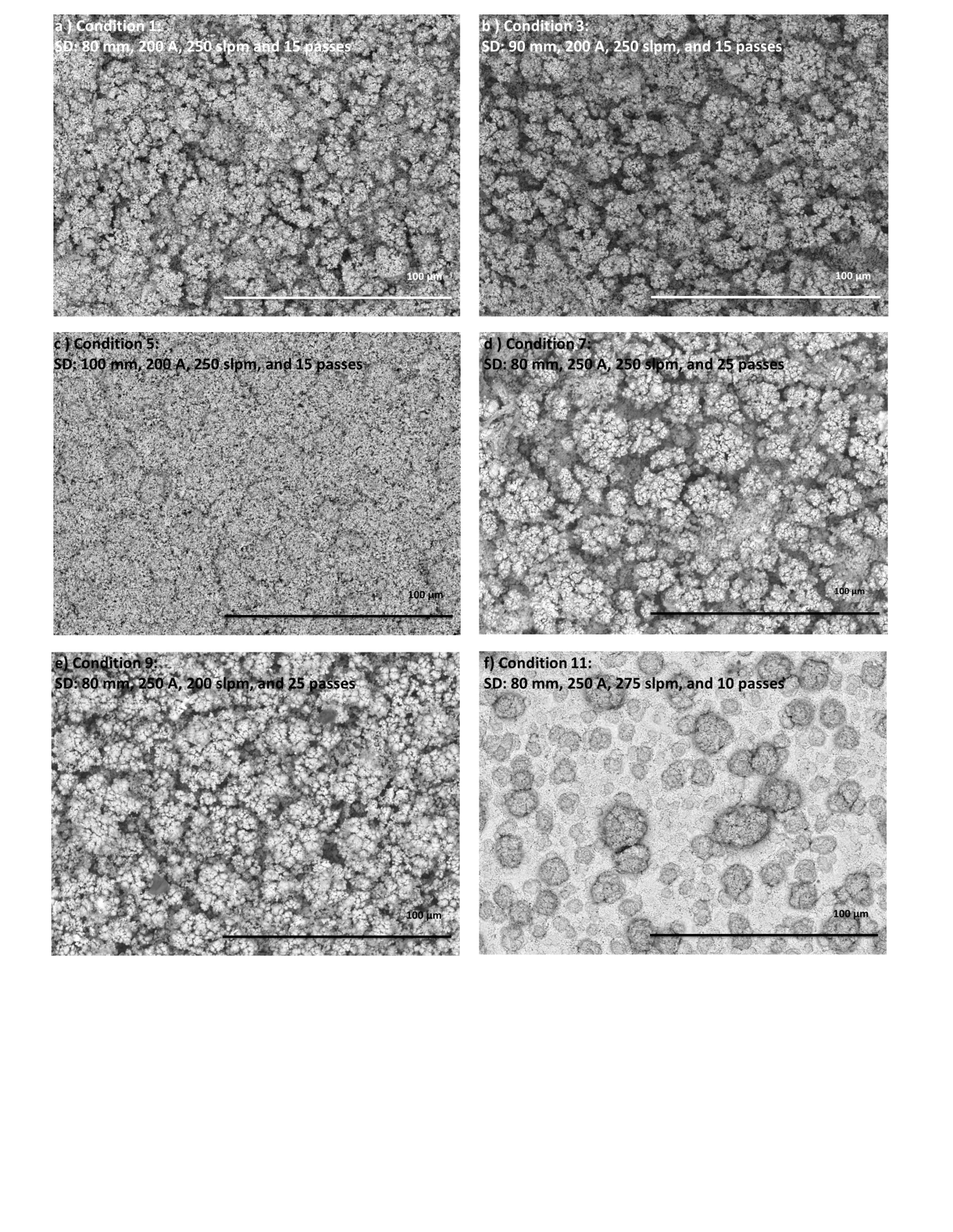


Figure S5: Surface topographies of the coatings.

**Supplementary Movies:**

Movie S1: Single water droplet bouncing off the coated surface. The water droplet size is ~ 2.5 mm and it has an impact velocity of ~ 1.5 m/s.

Movie S2: Water droplets coalescence on the coated surface. The size of the water droplets are ~ 2.5 mm and the second droplet has an impact velocity of ~ 1.5 m/s.
